# Human umbilical cord mesenchymal stem cells regulate immunoglobulin a secretion and remodel the diversification of intestinal microbiota to improve colitis

**DOI:** 10.3389/fcimb.2022.960208

**Published:** 2022-09-02

**Authors:** Airu Liu, Xing Wang, Xiaonan Liang, Wenxin Wang, Chenyang Li, Jiaming Qian, Xiaolan Zhang

**Affiliations:** ^1^ Hebei Key Laboratory of Gastroenterology, Hebei Clinical Research Center for Digestive Diseases, Department of Gastroenterology, Hebei Institute of Gastroenterology, The Second Hospital of Hebei Medical University, Shijiazhuang, China; ^2^ Department of Gastroenterology, Peking Union Medical College Hospital, Chinese Academy of Medical Sciences and Peking Union Medical College, Beijing, China

**Keywords:** mesenchymal stem cells, intestinal microbiota, colitis, immunoglobulin A, 16S rRNA

## Abstract

**Background:**

Mesenchymal stem cell (MSC) therapy has emerged as a promising novel therapeutic strategy for managing inflammatory bowel disease (IBD) mainly *via* dampening inflammation, regulating immune disorders, and promoting mucosal tissue repair. However, in the process, the associated changes in the gut microbiota and the underlying mechanism are not yet clear.

**Methods:**

In the present study, dextran sulfate sodium (DSS) was used to induce colitis in mice. Mice with colitis were treated with intraperitoneal infusions of MSCs from human umbilical cord mesenchymal stem cells (HUMSCs) and evaluated for severity of inflammation including weight reduction, diarrhea, bloody stools, histopathology, and mortality. The proportion of regulatory T cells (Tregs) and immunoglobulin A-positive (IgA^+^) plasmacytes in gut-associated lymphoid tissue were determined. The intestinal and fecal levels of IgA were tested, and the proportion of IgA-coated bacteria was also determined. Fecal microbiome was analyzed using 16S rRNA gene sequencing analyses.

**Results:**

Treatment with HUMSCs ameliorated the clinical abnormalities and histopathologic severity of acute colitis in mice. Furthermore, the proportion of Tregs in both Peyer’s patches and lamina propria of the small intestine was significantly increased. Meanwhile, the proportion of IgA^+^ plasmacytes was also substantially higher in the MSCs group than that of the DSS group, resulting in elevated intestinal and fecal levels of IgA. The proportion of IgA-coated bacteria was also upregulated in the MSCs group. In addition, the microbiome alterations in mice with colitis were partially restored to resemble those of healthy mice following treatment with HUMSCs.

**Conclusions:**

Therapeutically administered HUMSCs ameliorate DSS-induced colitis partially *via* regulating the Tregs–IgA response, promoting the secretion of IgA, and facilitating further the restoration of intestinal microbiota, which provides a potential therapeutic mechanism for HUMSCs in the treatment of IBD.

## Introduction

Inflammatory bowel disease (IBD), including Crohn’s disease and ulcerative colitis, is characterized by chronic intestinal inflammation, a progressive and unpredictable disease course. Although the pathogenesis of IBD has not been fully understood, intestinal dysbiosis is considered to be an important trigger for impaired intestinal barrier function and immune homeostasis, which may be a decisive event in the development and chronicity of IBD (Miyoshi and Chang, 2017). The composition of the microbiota in patients with IBD has been extensively studied. Multiple lines of evidence have revealed the presence of dysbiotic microbiota, characterized by decreased community diversity and a shift in bacterial taxa, including a decrease in certain genera of the phylum *Firmicutes* and *Bacteriodetes* and an increased abundance of Proteobacteria (Sartor and Wu, 2017). Schaubeck et al. (2016) demonstrated that transfer of fecal microbiota from mice with colitis to healthy animals induced colitis, and transplanting fecal flora from healthy people to mice with experimental colitis significantly improved the immune-inflammatory state of mice (Burrello et al., 2018). Therefore, restoring intestinal microbiota and improving the intestinal microecology have important clinical significance for the rescue treatment of IBD patients. Currently, therapies based on correcting dysbiosis, such as antibiotics, probiotics, and prebiotics, have shown certain auxiliary effects, but the effects are not definitive (Caruso et al., 2020). As an emerging microecological therapy, fecal microbiota transplantation also brings certain prospects for IBD patients, but the long-term tolerance and safety are still unclear (Weingarden and Vaughn, 2017). Thus, how to effectively reshape the intestinal flora is still an important issue in the field of IBD treatment.

Mesenchymal stem cell (MSC) therapy has emerged as a promising new therapeutic strategy for managing IBD mainly *via* dampening inflammation, regulating immune disorders, and promoting mucosal tissue repair. To date, most studies of MSC therapy for IBD have focused on immune modulation, promoting the proliferation and differentiation of T regulatory cells (Tregs), inhibiting the activity of inflammatory T cells, and the secretion of pro-inflammatory factors to maintain intestinal immune homeostasis and improve the mucosal inflammatory response (Shi et al., 2018). Recent studies found that MSCs not only improved the intestinal inflammatory response but also reshaped the diversity and abundance of intestinal flora, with a similar composition of bacterial taxa in mice with colitis to that of normal mice (Soontararak et al., 2018; Dong and Feng, 2019). Although sufficient evidence is still absent and the related mechanisms have not been reported, it is speculated that remodeling the intestinal microbiota may be another potential approach for MSCs in the treatment of IBD.

Currently, intravenous injection is historically the most common method for MSC delivery (Kean et al., 2013), and many published studies have shown the benefits of tail vein delivery in IBD treatment. However, an increasing body of evidence suggests that intraperitoneal injection (1 × 10^6^ cells) showed better colitis recovery, higher MSC engraftment at the inflamed colon but fewer trapped cells in the lung, and also more infiltration of Tregs (Sala et al., 2015; Wang et al., 2016) compared with intravenous injection. In addition, most of the current studies on the regulation of intestinal flora by MSCs apply tail vein intervention (Mar et al., 2014; Soontararak et al., 2018), while the effect of intraperitoneal injection with MSCs on gut microbiota is rarely reported. Thus, clarifying the influence of MSC intraperitoneal therapy on IBD intestinal flora and its possible mechanism will provide an important theoretical basis for its effective application in IBD.

It has been confirmed that the immune regulation of the host intestinal mucosa plays a crucial role in the maintenance of intestinal flora diversity (Sun et al., 2015). Tregs expressing transcription factor Foxp3 (Foxp3^+^Tregs) were essential for the maintenance of immune tolerance (Visekruna et al., 2019; Clough et al., 2020). It was found that the presence of Foxp3^+^Tregs in intestinal mucosa supported the transformation of IgA^+^ plasmacytes, maintaining the selective secretion of IgA in the gut and strengthening the immune barrier, although the mechanisms have not been completely clarified (Cong et al., 2009; Neumann et al., 2019). IgA has been shown to be involved in host responses against infection, and the major role of IgA is to maintain the balance between the host and its microbiota (Okai et al., 2017). Secretory IgA (SIgA) has been shown to play multiple protective roles by preventing the adhesion of commensal bacteria to epithelial cells, neutralizing toxins and pathogens, and limiting bacterial growth and penetration (Okai et al., 2017). It was found that, in a T cell transfer colitis model, the transfer of Foxp3^+^Tregs could inhibit the inflammatory response of the colon, induce the transformation of IgA^+^ plasmacytes in the lamina propria of the intestinal mucosa, promote the production of SIgA, and maintain the diversity of gut microbiota (Kawamoto et al., 2014; Wang et al., 2015), while depletion of Foxp3^+^Tregs decreased the intestinal IgA responses, resulting in a decrease in firmicutes and an increase in proteobacteria and aggravating colitis (Cong et al., 2009; Kawamoto et al., 2014). Therefore, regulating the intestinal IgA responses by Foxp3^+^Tregs, also called Tregs–IgA response, assumes an important role in maintaining the intestinal microecology (Cong et al., 2009).

Given the important role of Foxp3^+^Tregs, whether the mechanism of MSC remodeling gut microbiota is related to the regulation of intestinal Tregs–IgA response deserves further exploration. Therefore, in the present study, we investigated the effectiveness of human umbilical cord mesenchymal stem cells (HUMSCs) for the treatment of IBD and the influence on gut microbiota using a mouse model of DSS-induced colitis. Studies were also done to first investigate the possible mechanisms by which MSCs restore the gut microbiota in IBD. The findings revealed that HUMSCs restored the gut microbiota at least partly through the regulation of the intestinal Tregs–IgA response to accelerate the recovery of intestinal abnormalities in mice with colitis.

## Materials and methods

### Cell preparation and culture

Human umbilical cord mesenchymal stem cells provided by Shandong Qilu Cell Therapy Engineering Technology Co., Ltd. were cultured in a serum-free MSC medium (Yocon Biology, Beijing, China) at 37°C in a 5% CO_2_ incubator. The phenotype of the MSCs was identified through flow cytometry which examined the expression of cell surface markers, including positive markers CD90, CD105, CD73, CD44, and CD29 and negative markers CD45 and HLA-DR. HUMSCs from passages 4 and 7 were used throughout the experiments.

### Animals

Eight-week-old wild-type specific pathogen-free male C57BL/6 mice (weight, 20–23 g; Beijing Vital River Laboratory Animal Technology Co., Ltd.) were used for the induction of colitis. The mice were housed in a specific pathogen-free animal laboratory with an environment that has a constant temperature of 23°C ( ± 2°C) and alternating 12-h light/12-h dark cycle. All animal experiments were approved by the Local Animal Ethics Committee.

### Colitis mouse model

Colitis was induced in mice by the oral administration of 2% dextran sodium sulfate (DSS) (MP Biomedicals, USA) in drinking water for 7 days as we described previously (Yang et al., 2021). For each study, mice (*n* = 6 per group) were randomly assigned to the following groups: (a) control group, (b) DSS+PBS group, and (c) DSS+MSCs group. On day 5 of the study (with DSS administration initiated on day 0), MSCs were administered by peritoneal injection at a dose of 1 × 10^6^ cells per mouse in 200 ul phosphate-buffered saline (PBS) according to the previous study (Soontararak et al., 2018). The control and DSS groups of mice were administered with 200 ul PBS by peritoneal injection. At 7 days later, the mice were administered with drinking water without DSS and euthanized on day 10 of the study.

### Assessment of colitis

For each study, the mice were checked daily for morbidity. Pathological features, including stool consistency, presence of blood stool, and body weight loss, were recorded daily for each mouse. Individual scores were combined to generate the Disease Activity Index (DAI) in assessing disease severity as described previously (Yang et al., 2021). Colon tissue samples were collected, fixed in formalin, and then stained with hematoxylin and eosin (H&E) for histopathological analysis to evaluate the severity of histological damage of colitis using the Cooper HS score system (Yang et al., 2021).

### Fecal genomic DNA extraction

The fecal samples were harvested under sterile conditions, immediately shock-frozen in liquid nitrogen, and then transferred to -80°C. The CTAB/SDS method was used to extract the total genome DNA in samples as described previously (Wang et al., 2020). DNA concentration and purity were monitored on 1% agarose gels. According to the concentration, DNA was diluted to 1 ng/µl with sterile water.

### 16S rRNA sequencing

The 16S rRNA genes of V4 region were amplified with specific primer V4: 515F-806R. All PCR mixtures contained 15 µl of Phusion^®^ High-Fidelity PCR Master Mix (New England Biolabs), 0.2 µM of each primer, and 10 ng target DNA, and the cycling conditions consisted of a first denaturation step at 98°C for 1 min, followed by 30 cycles at 98°C (10 s), 50°C (30 s), and 72°C (30 s) and a final 5-min extension at 72°C. The PCR products were purified with Qiagen Gel Extraction Kit (Qiagen, Germany). Following the manufacturer’s recommendations, sequencing libraries were generated with NEBNext^®^ Ultra™ IIDNA Library Prep Kit (catalogue number E7645) and sequenced on an Illumina HiSeq 2500 platform, and 250-bp paired-end reads were generated. The paired-end reads were truncated by cutting off the barcodes and primer sequences, and then high-quality Clean Tags were obtained through splicing and filtering. The Clean Tags were compared with the reference database (Silva database http://www.arb-silva.de/). The Effective Tags were denoised with DADA2 in the QIIME2 software (Version QIIME2-202006) to obtain the initial amplicon sequence variants (ASVs), and then ASVs with abundance less than 5 were filtered out. The absolute abundance of ASVs was normalized using a standard of sequence number corresponding to the sample with the least sequences. Subsequent analyses of alpha diversity and beta diversity were all performed based on the output normalized data. For alpha diversity analysis, Chao1, Simpson, and Pielou_e indexes for each group were determined using QIIME2 software. The bacterial community difference among different groups was evaluated with principal coordinate analysis (PCoA) and non-metric multi-dimensional scaling (NMDS) based on weighted UniFrac distances. The LEfSe software (version 1.0) was used to do LEfSe analysis (linear discriminant analysis score threshold: 4) so as to find out the biomarkers.

### Mononuclear cell isolation

Cells from intestinal lamina propria, Peyer’s patches, and mesenteric lymph nodes were isolated as described previously (Rios et al., 2016). Briefly, Peyer’s patches and mesenteric lymph nodes were mechanically dissociated in ice-cold PBS. The resulting cell suspensions were passed through a 70-um mesh cell strainer. To prepare the intestinal lamina propria cells, associated fat and Peyer’s patches were removed, the intestinal tissue was washed in ice-cold PBS to remove the luminal contents and cut open longitudinally, and the tissue was cut into four equal-sized pieces. Epithelial cells were removed by shaking the tissues in PBS with 1 mM EDTA, 1 mM dithiothreitol, and 10% fetal calf serum for two rounds of 20 min at 37°C. Then, the pieces were washed three times with PBS to remove the EDTA, minced exactly 40 times in a microfuge tube, and incubated in 20 ml of RPMI-1640 supplemented with 1.5 mg ml^-1^ Collagenase II (Biosharp), 2.5 mg ml^-1^ hyaluronidase (Biosharp), and 0.25 mg ml^-1^ DNase I (Solarbio) for 45 min at 37°C with constant shaking. Cell suspension was then extracted by passing the tissue and supernatant over a 70-µm mesh cell strainer. The cell suspension was then centrifuged, and the resuspended pellet was further purified from the interface of a 45/72% Percoll density gradient.

### Flow cytometry analysis

Single-cell preparations with one million cells per 100 ul PBS were stained with antibodies to the following markers: APC/Cy7-anti-mouse CD4 (100413; Biolegend), PE/Cy7-anti-mouse CD25 (102015; Biolegend), APC-anti-mouse B220 (103221; Biolegend), PE-anti-mouse CD45 (103105-50; Biolegend). For intracellular staining, all cells were fixed for 40 min in BD Fix/Perm buffer and then washed in BD Perm/Wash buffer. The cells were then stained with PE-anti-mouse FOXP3 (126403; Biolegend) and FITC-anti-mouse IgA (11-4204-81; Biolegend) for 30 min at 4°C. Stained cells were tested on a FACSVerse flow cytometer (BD Biosciences, San Jose, CA, USA) and analyzed using FlowJo software (TreeStar, USA).

### Evaluation of IgA-coated bacteria by flow cytometry

Fecal pellets were freshly harvested and incubated in sterile PBS (100 µl to 10 mg feces) for 1 h at 4°C, homogenized, and centrifuged at 600 g for 5 min to remove large particles. Supernatant was centrifuged at 15,000*g* for 5 min to remove non-bound immunoglobulins. The bacteria pellet was resuspended in sterile bovine serum albumin/PBS (1% w/v). The bacteria were stained with FITC-anti-mouse IgA for 30 min on ice and washed with sterile PBS twice. The stained bacteria were tested on a FACSVerse flow cytometer (BD Biosciences, San Jose, CA, USA) and analyzed using FlowJo software (TreeStar, USA).

### Fecal IgA ELISA

Fecal pellets were freshly harvested and incubated in sterile PBS (100 µl to 10 mg feces) for 1 h at 4°C, homogenized, and centrifuged at 600*g* for 5 min to remove large particles. Supernatant was centrifuged at 15,000*g* for 5min to separate the bacterial pellet, and IgA was evaluated using the Mouse IgA ELISA Kit from MultiSciences according to the manufacturer’s instructions.

### Immunohistochemical analysis

The levels of IgA in intestinal tissues were determined by immunohistochemical staining. Mice intestinal tissues were fixed in formalin and embedded in paraffin. The paraffin-embedded tissues were cut into 4-μm sections for immunostaining. The sections were hatched with anti-IgA (11449-1-AP; Proteintech) primary antibodies. Next, the sections were incubated with a secondary antibody using the SP (Rabbit) IHC Kit (SP9001; ZSGB-BIO) following the manufacturer’s instructions. Optical microscopy was used for observation, and the expression of IgA was analyzed using ImageJ.

### Statistical analysis

Data were analyzed using GraphPad Prism version 8.0 (GraphPad Software, San Diego, CA, USA), and the results were shown as mean ± SD. Differences between the mean values for groups were analyzed by an unpaired two-tailed Student’s *t*-test. Comparisons of parameters for three groups were made by one-way analysis of variance (ANOVA) followed by Tukey’s test. The intestinal microbiota data were analyzed using the QIIME2 software of Novogene Biotech Co., Ltd., Beijing, China. A value of *P <*0.05 was considered statistically significant.

## Results

### Phenotypic characterization of HUMSCs

**Figure 1:**
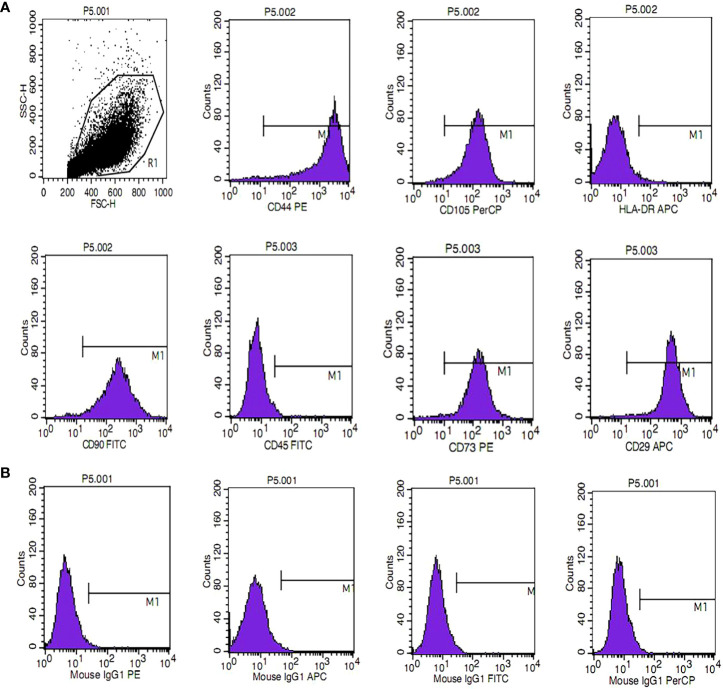
Phenotypic characterization of human umbilical cord mesenchymal stem cells. **(A)** The phenotype of mesenchymal stem cells (MSCs) was identified, including positive markers CD90, CD105, CD73, CD44, and CD29 and negative markers CD45 and HLA-DR. **(B)** Isotype antibodies were used as controls.

HUMSCs were assessed for MSC characteristics by harvesting cells at the third passage and analyzing them by flow cytometry. As shown in **Figure 1**, the cells were positive for the expression of CD90, CD105, CD29, CD44, and CD73 but negative for the expression of CD45 and HLA-DR. The results showed that the cells were HUMSCs and could be used for subsequent experiments.

### HUMSCs attenuated DSS-induced colitis in mice

**Figure 2:**
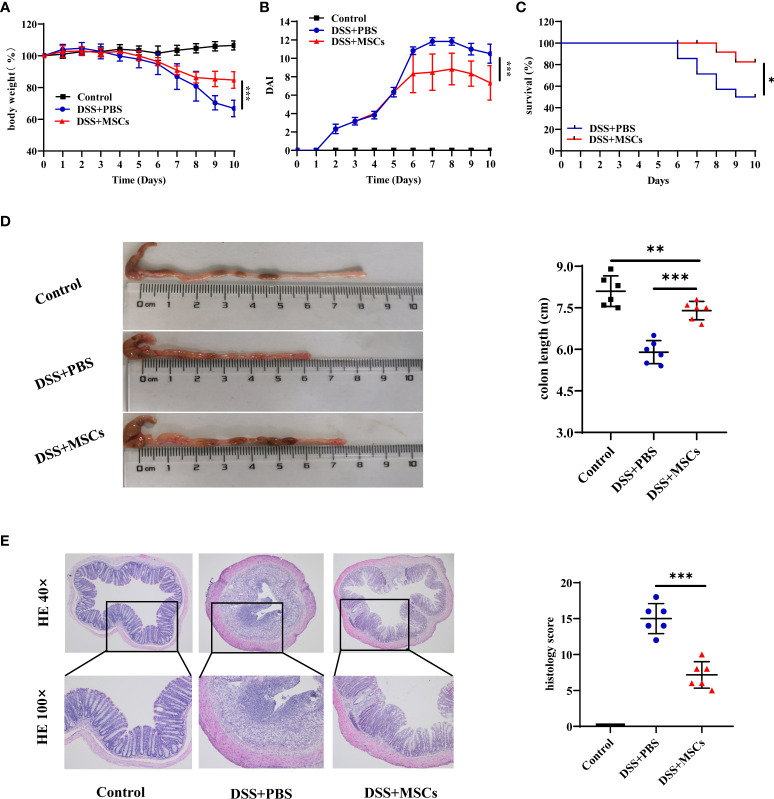
Mesenchymal stem cells (MSCs) from human umbilical cord ameliorated dextran sulfate sodium (DSS)-induced experimental colitis. The mice were treated with 2.0% DSS in their drinking water for 7 days, followed by 3 days of normal water. MSCs suspended in phosphate-buffered saline (PBS) were administered by intraperitoneal injection in the DSS+MSCs group on day 5, while the DSS+PBS group was only intraperitoneally injected with the same volume of PBS. **(A)** Body weight change (*n* = 6). **(B)** Disease activity index (DAI) score (*n* = 6). **(C)** Survival (*n* = 14). **(D)** Representative pictures of colon and colon length (*n* = 6). **(E)** Representative microscopic pictures of H&E staining (×40 and ×100 magnification) and histopathological score (*n* = 6). The results were reported as mean ± SD. *P*-values were calculated by one-way analysis of variance followed by Tukey’s test; *p < 0.05, **p <0.01, ***p < 0.001.

To investigate whether MSCs have a therapeutic effect on IBD, DSS-induced colitis in a mouse model was employed. Mice were treated with 2.0% DSS in their drinking water for 7 days followed by 3 days of normal water. MSCs suspended in PBS were administered by intraperitoneal injection in the DSS+MSCs group on day 5, while the DSS+PBS group was only intraperitoneally injected with the same volume of PBS. Compared with the DSS+PBS group, MSC administration significantly ameliorated DSS-induced colitis as evidenced by the marked restoration of weight loss (**Figure 2A**), decreased mortality (**Figure 2B**), and significant relief of colonic shortening (**Figure 2C**). Furthermore, the DAI based on the assessment of stool consistency, bloody stool, and body weight loss displayed a consistent tendency after MSC administration (**Figure 2D**). H&E staining was performed to systematically evaluate the severity of colonic mucosa injury. Compared with the DSS+PBS group which presented with more loss of crypts, infiltration of mononuclear cells, severer damage of goblet cells, and higher histopathological score, the DSS+MSCs group exhibited a relatively intact colonic architecture, less mononuclear cell infiltration, mild mucosal damage, and a lower histopathological score (**Figure 2E**).

### Administration of HUMSCs reshaped the diversity and richness of gut microbiota

To determine whether the repairing effect of MSCs on colon inflammation is related to the regulation of gut microbiota, 16S rRNA sequencing analysis was performed in fecal bacterial DNA isolated from the control group, the DSS+PBS group, and the DSS+MSCs group of mice.

**Figure 3:**
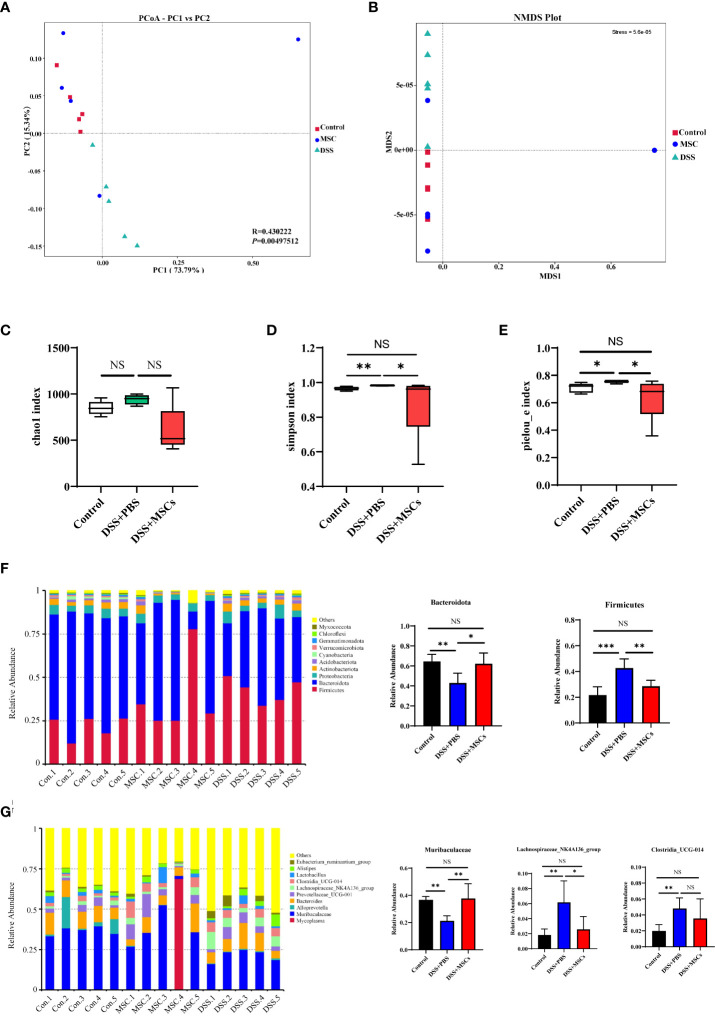
Human umbilical cord mesenchymal stem cell treatment significantly reversed the microbiome dysbiosis induced by dextran sulfate sodium drinking. **(A)** Principal coordinate analysis based on weighted UniFrac distances for beta diversity; the differences were evaluated by anosim analysis. **(B)** Non-metric multi-dimensional scaling based on weighted UniFrac distances for beta diversity. Alpha diversity box plot of chao1 index **(C)**, Simpson index **(D)**, and Pielou_e index **(E)**. Histograms of the relative abundance for the top 10 microbes at the phylum **(F)** and genus **(G)** levels. *P*-values were calculated by ANOVA, followed by Tukey’s test. The results were expressed as mean ± SD; *n* = 5, *p < 0.05, **p <0.01, ***p < 0.001. NS, *p* > 0.05.

First, to evaluate the complexity of the community composition and compare the differences between groups, beta diversity was calculated based on weighted UniFrac distances. Both models of PCoA and NMDS were performed to visualize differences of samples in complex multi-dimensional data. As shown in **Figures 3A, B**, the difference of microflora structure among the three groups was obvious, but compared with the DSS+PBS group, the community composition was relatively similar between the control mice and the MSC mice.

To evaluate the differences of community distribution within the groups, alpha diversity was calculated. The chao1 index represents community richness. As shown in **Figure 3C**, the number of intestinal flora in the DSS+PBS group was higher than that of the control group, and MSC treatment decreased the number of intestinal flora despite the fact that no statistical difference was achieved. The indexes of Simpson and Pielou_e were measured for microbial community diversity and evenness. Compared with the control group, both alpha diversity indices were significantly upregulated with DSS administration, while MSC treatment reversed the change, and there was no difference between the MSCs group and the control group (**Figures 3D, E** **)**.

Next, we analyzed the difference of populations and abundances among the three groups. The top 10 microbes at the phylum, class, order, family, and genus levels were shown and indicated significant variations in the composition of the gut microbiota. Analyses of the microbiota at the phylum level revealed a dominance of *Firmicutes* and *Bacteroidota*. Compared with the control mice, DSS administration significantly increased *Firmicutes* and decreased *Bacteroidota*, and MSC treatment reversed this change, decreasing *Firmicutes* and increasing *Bacteroidota* (**Figure 3F**). At the genus level, the *Muribaculaceae* bacteria community in the DSS+PBS group was significantly lower than that in the Control group, and MSC treatment increased the community remarkably as shown in **Figure 3G**. Similarly, MSCs restored the change in *Lachnospiraceae_NK4A136_group* and *Clostridia_UCG-014* induced by DSS drinking (**Figure 3G**). Besides this, the effect of MSCs in restoring the microbial population was also observed at the class, order, and family levels (**Supplementary Figure S1**).

### HUMSC treatment restored the microbial structure in mice induced by DSS

**Figure 4:**
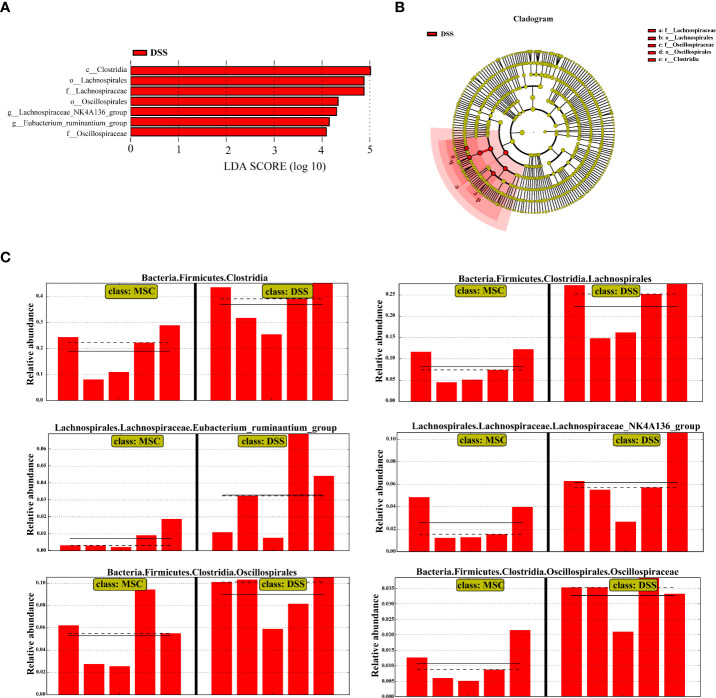
Human umbilical cord mesenchymal stem cell treatment restored the microbial structure in mice induced by dextran sulfate sodium (DSS). **(A)** Linear discriminant analysis (LDA) scores computed to identify the taxa which were significantly enriched in each group (LDA score > 4). There are no significantly different taxa in the DSS + mesenchymal stem cell group, so this group is not shown. **(B)** LEfSe cladogram representing the microbiota enriched in the DSS + phosphate-buffered saline (PBS) group. Nodes from inside out represent taxonomic types from phylum to genus levels. The sizes of the nodes indicate the relative abundance of the taxa. Red nodes denote the taxa which were significantly enriched in the DSS+PBS group. **(C)** All-against-all algorithm of LDA coupled with LEfSe.

To confirm which bacterium was altered by MSC treatment and, in turn, affected the disease progression against DSS-induced colitis, we performed LEfSe analysis to detect significant differences in the dominance of bacterial communities. As shown in **Figures 4A, B**, the class *Clostridia*, the order *Lachnospirales*, and the family *Lachnospiraceae*, the order *Oscillospirales* and the family *Oscillospiraceae*, and the genus *Lachnospiraceae_NK4A136_group* and *Eubacterium_ ruminantium_group* were the dominant differential bacteria resulting in gut microbiota dysbiosis in the DSS group, while these types of taxa were downregulated with MSC administration (**Figure 4C**).

### HUMSCs regulated the response of Foxp3^+^Tregs and IgA-secreting plasmacytes

**Figure 5:**
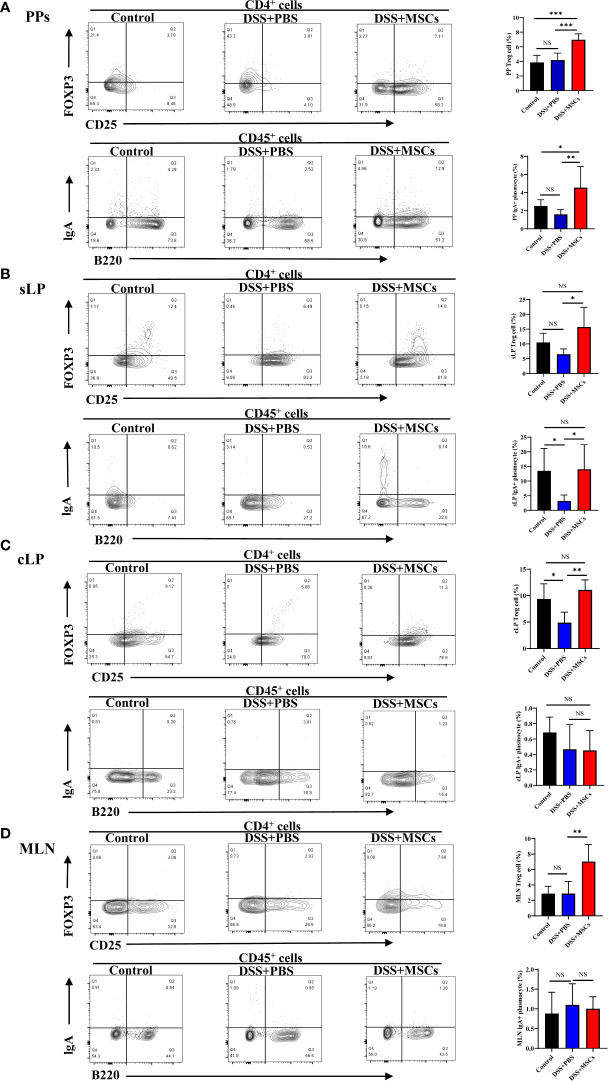
Human umbilical cord mesenchymal stem cell regulated the response of Foxp3^+^Tregs and IgA-secreting plasmacytes in Peyer’s patches (PPs) and lamina propria of the small intestine (sLP). CD4^+^CD25^+^Foxp3^+^ T cells (Tregs) and CD45^+^B220^-^IgA^+^ B cells (IgA^+^ plasmacytes) from the control group, the dextran sulfate sodium (DSS) + phosphate-buffered saline group, and the DSS + mesenchymal stem cell group were analyzed by flow cytometry, and bar charts of the percentage of Foxp3^+^Tregs and IgA^+^ plasmacytes in PPs **(A)**, sLP **(B)**, colonic lamina propria **(C)**, and mesenteric lymph nodes **(D)** are presented. The data are shown as mean ± SD; *n* = 4 to 5 per group. *P*-values were calculated by ANOVA, followed by Tukey’s test; *p < 0.05, **p <0.01, ***p < 0.001. NS, *p* > 0.05.

B220^-^IgA^+^ B cells are the mature plasmacytes secreting IgA, and Foxp3^+^Tregs have been shown as the important helper cells in affecting the transformation of IgA-secreting plasmacytes in GALT (Cong et al., 2009). Thus, to further explore whether the regulation of MSCs on intestinal flora was associated with Tregs–IgA response, we isolated mononuclear cells from GALT, including colonic lamina propria (cLP), small intestinal lamina propria (sLP), Peyer’s patches (PPs), and mesenteric lymph nodes (MLN) to determine the changes of Foxp3^+^Tregs and IgA^+^ plasmacytes by flow cytometry. Compared with the DSS+PBS group, the ratio of Foxp3^+^Tregs (CD4^+^CD25^+^Foxp3^+^T cells) was significantly enhanced with MSC treatment in PPs, and IgA^+^ plasmacytes (CD45^+^B220^-^IgA^+^ B cells) also displayed a higher percentage in PPs (**Figure 5A**). The consistent tendency was present in sLP after MSC administration (**Figure 5B**). Meanwhile, the DSS+MSCs group also had a higher absolute number of Foxp3^+^Tregs and IgA^+^ plasmacytes compared to the DSS group in PPs and sLP (**Supplementary Figure S2**), while in cLP, although MSC treatment markedly increased the ratio of Foxp3^+^Tregs, no difference was seen in IgA^+^ plasmacytes compared with the untreated group (**Figure 5C**). In addition, the proportions of Foxp3^+^Tregs and IgA^+^ plasmacytes in the MLN showed similar changes to those in cLP (**Figure 5D**). These data demonstrated that Tregs–IgA response was mainly present in PPs and sLP as previously evidenced (Kawamoto et al., 2014).

### HUMSCs promoted IgA production and IgA coating of microbiota in colitis mice

**Figure 6:**
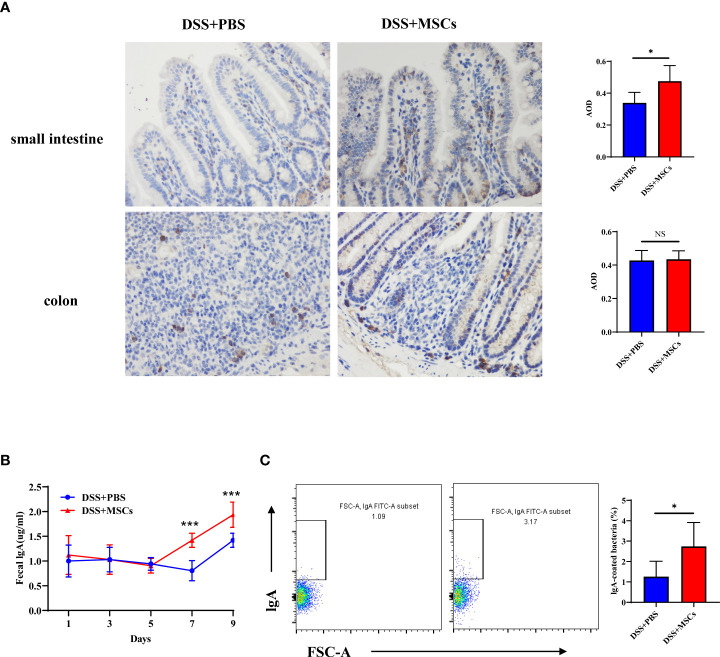
Human umbilical cord mesenchymal stem cell promoted IgA production and IgA coating of microbiota in colitis mice. **(A)** Expression of IgA in the small intestine and the colon from the dextran sulfate sodium (DSS) + phosphate-buffered saline (PBS) group and the DSS + mesenchymal stem cell (MSC) group by immunohistochemical staining (×400 magnification, positive for brown) and bar charts of average optical density values; *n* = 5 per group. **(B)** Levels of fecal IgA from DSS+PBS mice and DSS+MSCs mice on days 1, 3, 5, 7, and 9; *n* = 6 per group. **(C)** IgA coating of microbiota in fecal samples from the DSS+PBS group and the DSS+MSCs group was analyzed by flow cytometry, and bar charts of the proportion of IgA-coated bacteria are presented; *n* = 6. The data are shown as mean ± SD. *P*-values were calculated using unpaired *T*-test; *p < 0.05, **p <0.01, ***p < 0.001. NS, *p* > 0.05.

IgA is a crucial defensive factor directly contacting the microbiota and altering the community structure. To further observe the expression of IgA in intestinal tissues, immunohistochemical analysis was employed. Compared with the untreated group, the expression of IgA was significantly higher in the small intestine after MSC administration, while no difference was seen in the colon (**Figure 6A**), which was consistent with the results of flow cytometry. Next, we collected fresh stool from mice of the DSS group and the MSC-treated group on days 1, 3, 5, 7, and 9. Fecal pellets were incubated in sterile PBS (100 µl to 10 mg feces) and centrifuged to obtain the supernatant. ELISA was applied to determine the IgA concentration secreted into the intestinal lumen. Data showed that an intraperitoneal injection of MSCs increased IgA in stool pellets to a much greater extent than an injection of PBS did (**Figure 6B**). It is accepted that IgA can control the infection by coating the pathogenic bacteria. We next evaluated the bacteria-coating properties of IgA elicited with or without MSC intraperitoneal intervention. Therefore, we prepared suspensions of fecal bacteria and measured the amount of IgA bound to individual bacteria by flow cytometry with a labeled secondary anti-IgA monoclonal antibody. As expected, the proportion of IgA-coated bacteria significantly increased with MSC administration (**Figure 6C**).

## Discussion

MSC therapy has emerged as a promising therapeutic strategy for managing IBD mainly *via* dampening inflammation, regulating immune disorders, and promoting mucosal tissue repair. While there are few and conflicting reports on the effects of MSCs on gut microbiota regulation, Mar et al. (2014) showed that MSC-treated animals exhibited no significant differences in overall microbial community diversity compared with DSS animals. Several other studies indicated that MSC therapy significantly reversed the dysbiosis of DSS mice, remodeling the microbiota similar to that of the healthy group, although the possible mechanism has been not reported (Soontararak et al., 2018; Ikarashi et al., 2019). In addition, most of the current studies on the regulation of intestinal flora by MSCs apply tail vein intervention. In the present study, we found that, beyond improving colonic inflammation, MSC intraperitoneal injection also significantly promoted the remodeling of the gut microbiota in DSS-induced mice, which was probably related to the regulation of Tregs–IgA response.

In our study, C57BL/6 mice were administered with drinking water with DSS, and MSCs were administered by peritoneal injection on day 5. Previous reports have demonstrated that MSCs could reduce weight loss, colonic shortening, and histopathological score in IBD induced by DSS. In our current study, compared with the DSS group, MSC treatment significantly alleviated the DAI score, reduced the mortality, increased the colon length, and promoted the recovery of mucosal inflammation, which was consistent with the results of previous studies (Song et al., 2018).

The imbalance of the gut microbiota destroys immune homeostasis and triggers immune-mediated intestinal mucosal inflammation, which is widely considered as a key factor in the development of IBD (Miyoshi and Chang, 2017). Correction of dysbiosis is of great clinical significance for the treatment of IBD. In the present study, before euthanization, feces of mice were collected to perform 16S rRNA sequencing. We observed that the microbiome disruption induced by DSS injury was, to a great degree, reversed by the administration of MSCs as previously reported (Soontararak et al., 2018)—for example, compared to the DSS group, both the alpha and beta diversity and the abundance of the gut microbiome were significantly remodeled to become more closely resembling that of mice treated with MSCs in the healthy control group. LEfSe analysis also revealed several taxa with remarkable differential predominance in the DSS group, while MSC administration obviously reversed these changes. Therefore, although it is not clear whether the gut microbiota changes are the consequence of intestinal recovery or whether intestinal recovery results from the improved microbiota, these findings were sufficient to suggest that MSC administration accelerated overall intestinal health and healing through microbiome regulation.

However, the mechanism by which MSCs regulate the gut microbiota is unclear. Previous observations point to the existence of a Tregs–IgA axis in maintaining the balance of the gut microbiota (Feng et al., 2011; Kato et al., 2014; Luu et al., 2017). Foxp3^+^Tregs, by acting in both germinal center-independent and germinal center-dependent manners, suppress inflammation and support IgA^+^ plasmacyte transformation in PPs, resulting in an increase of SIgA, and their depletion causes a rapid loss of specific IgA response in the intestine (Cong et al., 2009). Current studies have shown that MSCs maintain intestinal mucosal immune homeostasis in IBD by promoting the proliferation and differentiation of Foxp3^+^Tregs in GALT (Shi et al., 2018), while the effect of MSCs on the transformation of IgA^+^ plasmacytes in the intestine is still unclear. In the present study, we examined the proportion of Foxp3^+^Tregs and IgA^+^ plasmacytes in GALT and observed that both the ratios and the numbers of Foxp3^+^Tregs and IgA^+^ plasmacytes were significantly upregulated with MSC treatment in PPs and sLP compared with the DSS group, while in cLP and MLN, although MSC treatment markedly enhanced the ratio of Foxp3+Tregs, the proportion of IgA^+^ plasmacytes had no difference compared with the untreated group. As previously reported, Tregs–IgA response was mainly present in PPs and sLP; Foxp3^+^ Tregs migrate into PPs, inducing the transformation of IgA^+^ plasmacytes which transfer to the sLP where they complete their differentiation and secrete IgA into the gut lumen (Kawamoto et al., 2014; Gribonika et al., 2019). These data supported the important role of MSCs in the regulation of the Tregs–IgA response. IgA is a crucial defensive factor directly contacting the microbiota and altering the community structure to strengthen the immune barrier. A previous study examined the potential of adipose-derived MSCs to restore the intestinal mucosal immune system in aged mice, which found that SIgA responses were significantly increased in aged mice adoptively transferred with MSCs when orally immunized with ovalbumin plus cholera toxin (Aso et al., 2016). In this study, we found that the level of IgA in the small intestine and feces was significantly upregulated after MSC treatment compared with the DSS group. Meanwhile, the proportion of IgA-coated bacteria significantly increased with MSC administration. It is widely accepted that IgA can control infection by coating the pathogenic bacteria and preventing their contact to the gut epithelium, a process called immune exclusion. Meanwhile, the coating by IgA regulates the diversity and the structure of the microbial taxa (Macpherson et al., 2018; Melo-Gonzalez et al., 2019). These findings suggest that MSCs remodeled the structure and the diversity of the gut microbiota by regulating the Tregs–IgA response, promoting intestinal IgA secretion.

This study is the first to explore the mechanism of MSCs regulating the gut microbiota. However, it should also be acknowledged that an in-depth investigation of its mechanism is lacking in the present study—for example, depleting Tregs to observe whether MSCs can still promote the transformation of IgA^+^ plasmacytes and the secretion of IgA to regulate the structure and the diversity of the intestinal flora and analyzing the IgA-coated bacteria with 16S rRNA sequencing. Therefore, there is still a need to further investigate and elucidate the mechanism by which MSCs regulate the gut microbiota.

## Conclusion

**Figure 7:**
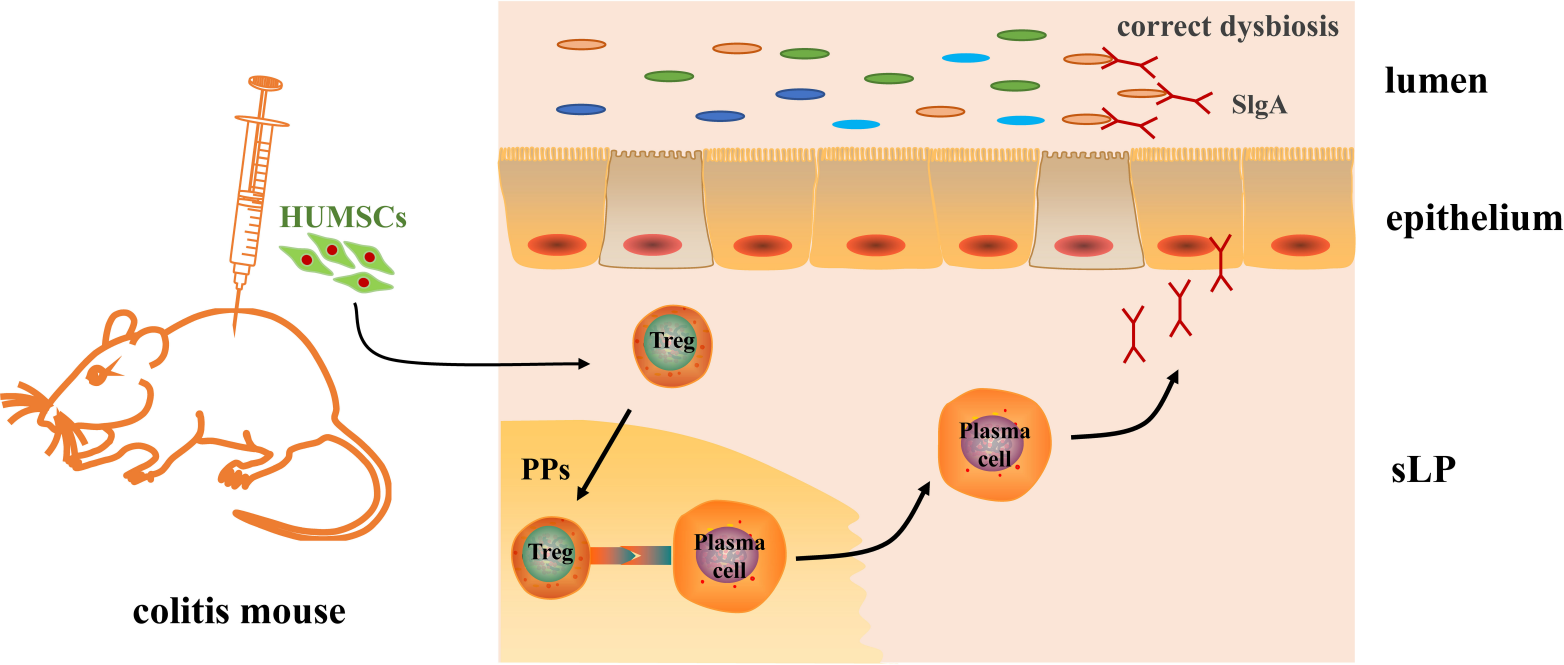
Mechanism of human umbilical cord mesenchymal stem cell (HUMSC)-regulated gut microbiota in dextran sulfate sodium-induced colitis mice. An intraperitoneal injection of HUMSCs regulated the intestinal Tregs–IgA response in Peyer’s patches, increased the IgA-secreting plasm cells in the lamina propria of the small intestine, and further promoted the secretion of IgA in the intestinal lumen, which contributed to the remodeling of the structure and the diversity of the gut microbiota.

Overall, our study here revealed that MSC intraperitoneal therapy improved DSS-induced colonic inflammation, and the mechanism may be partly by regulating the intestinal Tregs–IgA response, promoting the secretion of IgA in the intestinal lumen, and remodeling the structure and the diversity of the gut microbiota (**Figure 7**), which provides a potential therapeutic mechanism for HUMSCs in the treatment of IBD.

## Data availability statement

The datasets presented in this study can be found in online repositories. The names of the repository/repositories and accession number(s) can be found below: https://www.ncbi.nlm.nih.gov/bioproject/PRJNA843867.

## Ethics statement

The animal study was reviewed and approved by the Research Ethics Committee of the Second Hospital of Hebei Medical University.

## Author contributions

XZ and JQ made the conception and design. AL and XW completed the experiment and drafted the article. XL, WW, and CL did the data analysis. All authors contributed to the article and approved the submitted version.

## Conflict of interest

The authors declare that the research was conducted in the absence of any commercial or financial relationships that could be construed as a potential conflict of interest.

## Publisher’s note

All claims expressed in this article are solely those of the authors and do not necessarily represent those of their affiliated organizations, or those of the publisher, the editors and the reviewers. Any product that may be evaluated in this article, or claim that may be made by its manufacturer, is not guaranteed or endorsed by the publisher.
