# Supplementary material for: Human umbilical cord mesenchymal stem cells regulate immunoglobulin a secretion and remodel the diversification of intestinal microbiota to improve colitis
Source: Front Cell Infect Microbiol. 2022 Sep 2;12:960208. doi: 10.3389/fcimb.2022.960208 (PMC9478446; doi:10.3389/fcimb.2022.960208)
Supplement: Supplementary file 1 [file DataSheet_1.pdf]

Supplementary Material

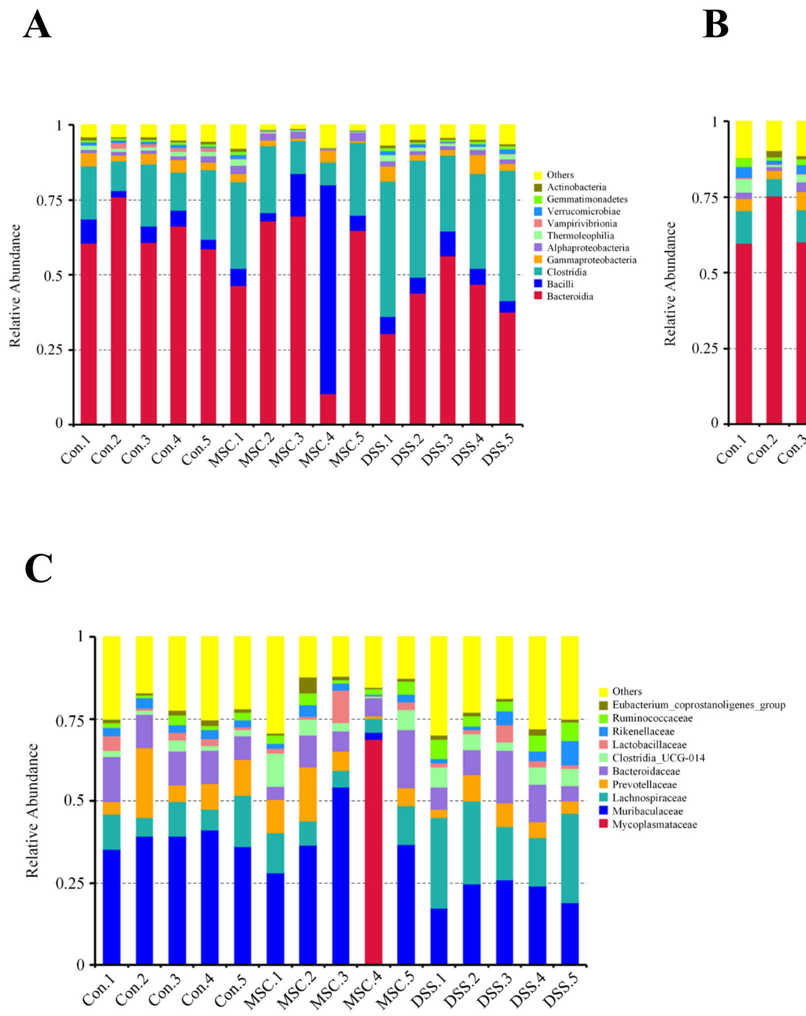

Supplementary Figure 1. HUMSCs treatment restored microbial population and abundance in mice induced with DSS. Histograms of relative abundance for top 10 microbes at the class (A), order (B) and family (C) level.

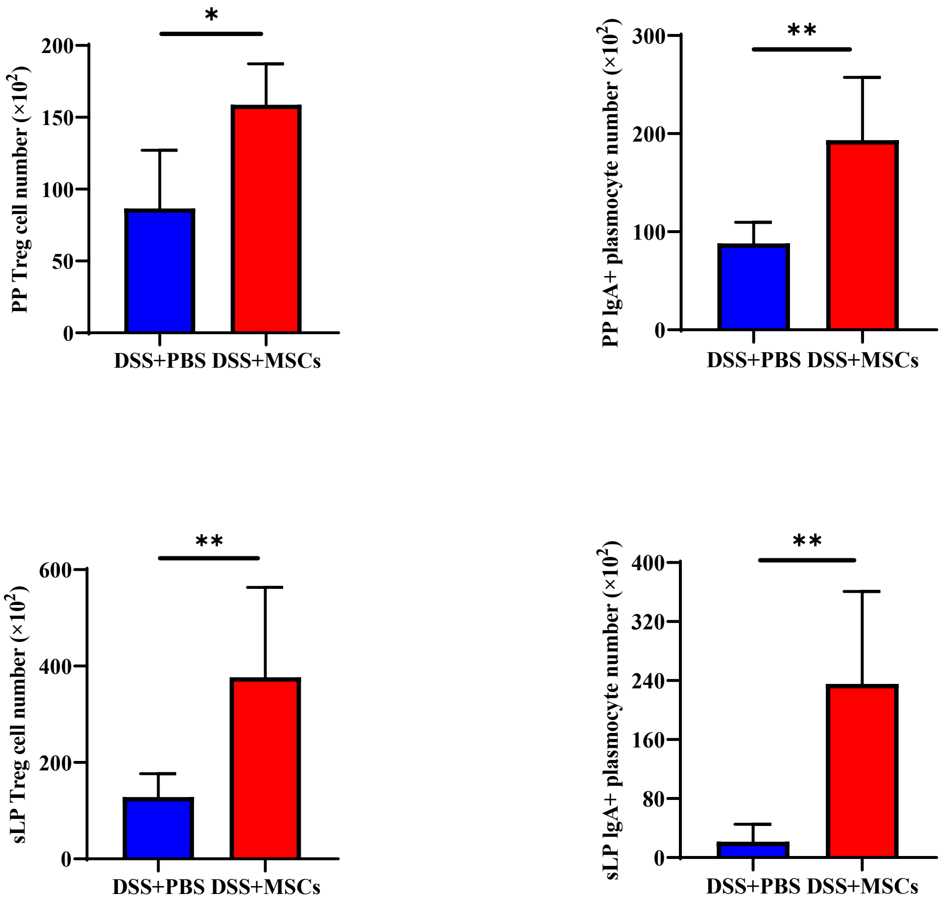

Supplementary Figure 2. HUMSCs upregulated the absolute number of Foxp3^{+}Tregs and lgA-secreting plasmacytes in PPs and sLP. Bar charts of the number of Foxp3^{+}Tregs and lgA^{+} plasmacytes in PPs (A) and sLP (B). Data were shown as mean ± SD; n=4-5 per group. *P* values were calculated by an unpaired two-tailed Student's t-test, * $p < 0.05$, ** $p < 0.01$, *** $p < 0.001$.
